# Unique role of DDX41, a DEAD-box type RNA helicase, in hematopoiesis and leukemogenesis

**DOI:** 10.3389/fonc.2022.992340

**Published:** 2022-09-02

**Authors:** Satoru Shinriki, Hirotaka Matsui

**Affiliations:** Department of Molecular Laboratory Medicine, Faculty of Life Sciences, Kumamoto University, Kumamoto, Japan

**Keywords:** acute myeloid leukemia, myelodysplastic syndromes, RNA helicase, DDX41, R-loop, RNA splicing, nucleic acid sensor

## Abstract

In myeloid malignancies including acute myeloid leukemia (AML) and myelodysplastic syndromes (MDS), patient selection and therapeutic strategies are increasingly based on tumor-specific genetic mutations. Among these, mutations in *DDX41*, which encodes a DEAD-box type RNA helicase, are present in approximately 2–5% of AML and MDS patients; this disease subtype exhibits a distinctive disease phenotype characterized by late age of onset, tendency toward cytopenia in the peripheral blood and bone marrow, a relatively favorable prognosis, and a high frequency of normal karyotypes. Typically, individuals with a loss-of-function germline *DDX41* variant in one allele later acquire the p.R525H mutation in the other allele before overt disease manifestation, suggesting that the progressive decrease in DDX41 expression and/or function is involved in myeloid leukemogenesis.RNA helicases play roles in many processes involving RNA metabolism by altering RNA structure and RNA-protein interactions through ATP-dependent helicase activity. A single RNA helicase can play multiple cellular roles, making it difficult to elucidate the mechanisms by which mutations in *DDX41* are involved in leukemogenesis. Nevertheless, multiple DDX41 functions have been associated with disease development. The enzyme has been implicated in the regulation of RNA splicing, nucleic acid sensing in the cytoplasm, R-loop resolution, and snoRNA processing.Most of the mutated RNA splicing-related factors in MDS are involved in the recognition and determination of 3’ splice sites (SS), although their individual roles are distinct. On the other hand, DDX41 is likely incorporated into the C complex of the spliceosome, which may define a distinctive disease phenotype. This review summarizes the current understanding of how DDX41 is involved in this unique myeloid malignancy.

## Introduction

Recent advances in comprehensive genomic analysis for malignancies including hematopoietic tumors has elucidated most of the driver gene mutations involved in the disease development or progression (1, 2). Analysis of a large number of samples has also led to the identification of low-frequency mutations that had previously been overlooked. With regard to hematopoietic malignancies, it is now clear that low-frequency germline mutations may drive pathology in tumors that were previously thought to arise *via* unknown mechanisms (3, 4). Based on these findings, the WHO classification of myeloid malignancies was updated in 2016 to introduce the concept of disease classification based on somatic and germline gene mutations (5). The discovery in 2015 that *DDX41* mutations are found in acute myeloid leukemia (AML) and myelodysplastic syndromes (MDS) is relevant to this revision. In brief, Polprasert et al. performed a comprehensive genetic analysis of families with suspected inherited myeloid malignancies without known mutations such as *RUNX1*, *CEBPA*, and *GATA2* and isolated *DDX41* as a new disease-associated gene (4). This was the first example of mutation of an RNA helicase-encoding gene in hematopoietic malignancies; the *DDX41* mutations are found in both MDS and *de novo* AML cases that does not exhibit non-hematopoietic phenotypes, and are generally characterized by the absence of marked thrombocytopenia before overt disease manifestation.

**Figure 1:**
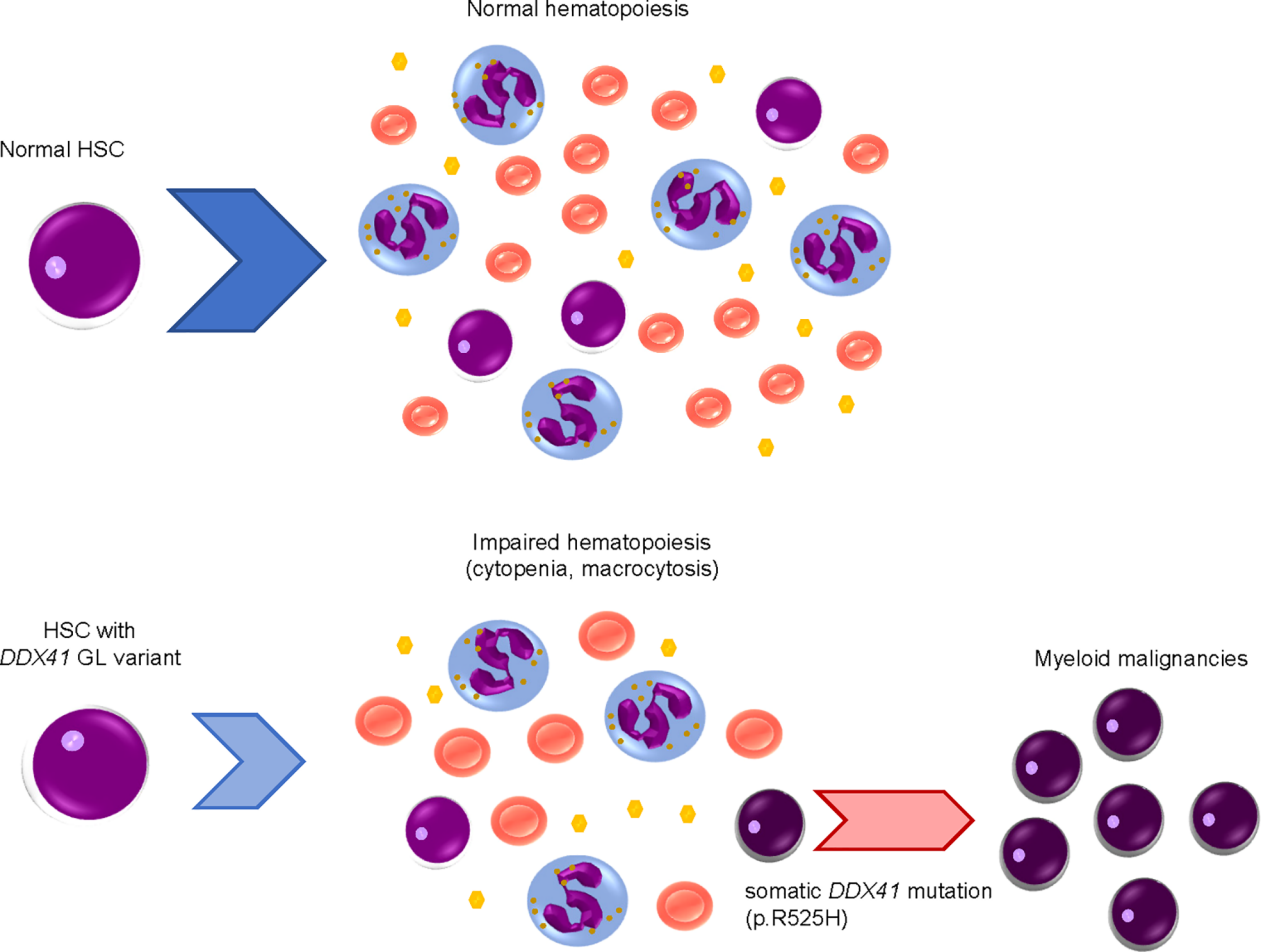
Development of myeloid malignancies by acquisition of DDX41 mutations in a stepwise manner.Hematopoietic stem cells (HSCs) with a heterozygous germline (GL) *DDX41* variant have mildly impaired hematopoiesis, including cytopenia and macrocytosis. Somatic *DDX41* mutation in the remaining wildtype allele later emerges within HSCs with a GL *DDX41* variant, which will lead to overt disease development.

As will be discussed later, *DDX41* encodes a DEAD-box type RNA helicase, which plays important roles in biological processes related to RNA metabolism. DDX41 performs these roles by converting RNA structure and changing interactions between RNA and proteins in an ATP-dependent manner (6–8). Several recent large clinical studies, including a prospective investigation, have established the clinical characteristics of myeloid malignancies with *DDX41* mutations (9–18). Of note, heterozygous germline *DDX41* variants cause mild defects in hematopoiesis; subsequent acquisition of a somatic mutation in the remaining wild-type allele at a different location from that of germline variants results in biallelic alteration, which is a requirement for a disease-developing driver mutation (**Figure 1**) (9, 10).

Although RNA helicase mutations have been reported sporadically in a number of malignancies, examples of driver mutations are quite limited. Two examples are mutation of *DHX15* in core binding factor leukemia (19, 20) and mutation or splice variants of *DHX34* in AML (3, 21). Largely however, the significance of *DDX41* mutations in disease pathogenesis remains poorly understood. In this review, we will present the clinical features of hematopoietic malignancies associated with *DDX41* mutations and discuss the molecular functions of DDX41.

## Clinical features of myeloid malignancies with *DDX41* mutations

*DDX41* mutations occur at a rate of 2 to 5% in AML (9, 10). Most affected patients are in their 60s and are therefore not markedly different to non-*DDX41* mutant sporadic AML cases with regard to peak age of disease diagnosis. The male-to-female ratio is around 3:1, suggesting a male predominance of the disease (22), but the reason for this is unclear. There does not appear to be a bias toward a specific AML subtype, but the disease is often characterized by low peripheral WBC counts and bone marrow hypoplasia, and a less differentiated tumor cell phenotype. However, there have been no reports of specific mutations in other genes that could contribute to these features of *DDX41* mutant AML (9). *DDX41* mutations are also observed in acute erythroid leukemia and lymphoid malignancies (23–25). Although solid tumors are sometimes found in patients with hematological malignancies and germline *DDX41* variants (26), an association between *DDX41* variants and onset of non-hematological maligancies has not been definitively shown.

As mentioned in the introduction, individuals with a germline *DDX41* variant acquire a somatic mutation before overt disease manifestation. The exact rate at which individuals with germline variants develop hematopoietic malignancies is still uncertain. However, 50–88% of MDS/AML patients with germline *DDX41* variants develop disease with somatic mutation (18). The fact that somatic *DDX41* mutations are the most frequent concomitant mutation with germline *DDX41* variants demonstrates that *DDX41* alterations are clearly linked to the disease etiology. The most frequent germline variant in the gene is p.D140fs, followed by p.M1I (27). Although the genomic positions at which germline variants occur may vary by race (28–31), the wide range of nonsense and frameshift mutations, especially in the N-terminal portion of the gene, strongly suggests that germline *DDX41* variants are loss-of-function type mutations. On the other hand, somatic mutations are highly concentrated in p.R525H, and less prevalently in p.G530D (14, 15). These somatic mutations are located within the helicase domain where ATP interacts with DDX41 (32), suggesting that somatic mutations interfere with the ATP-dependent helicase activity of the enzyme. Indeed, our previous study showed lower ATPase activity of the helicase domain with the p.R525H mutation (33). The reasons underlying the differential position of germline variants versus somatic mutations are not clear. However, the p.R525H mutation likely generates a hypomorphic protein that retains RNA-binding activity but has low helicase activity, which inhibits RNA and RNA/protein conformational conversion. Individuals with a germline variant sometimes manifest cytopenia and macrocytosis in the peripheral blood, and are thus likely to be diagnosed with idiopathic cytopenia of undetermined significance (ICUS) (34). This suggests that a 50% reduction of DDX41 expression or function affects hematopoiesis to some degree, but that this level does not impair the enzyme sufficiently to cause myeloid malignancies.

Recently, a large prospective study for AML with germline *DDX41* variants revealed that the response to conventional chemotherapy for the patients is relatively good, although relapse at 3 years post-treatment is comparable to that of patients with wild-type DDX41 (18). In addition, germline testing for *DDX41* before conducting allogeneic hematopoietic stem cell transplantation (27) should be conducted to reduce the potential risk of donor-derived leukemia (35–38).

As will be discussed later, AML cells with *DDX41* mutations tend to display an excessive DNA damage response, which may be due to the accumulation of DNA replication stress. On this basis, treatment of the disease with ATR inhibitors has been suggested (39), but to our knowledge, no clinical trials have yet been conducted. A few case reports suggested that lenalidomide may be efficacious in MDS with *DDX41* mutations, which could be related to the localization of *DDX41* in chr.5q35, which is likely to be deleted in the 5q- subset of patients (40, 41). However, no clinical trials have been conducted to test this hypothesis, possibly due to the relatively small number of patients available.

## Molecular function of DDX41

‘Helicase’ is the general term for enzymes that alter the tertiary structure of nucleic acids (both DNA and RNA) and proteins in this class are categorized into the SF1 to SF6 superfamilies (42). The SF2 superfamily is the largest, and contains the DEAD-box type RNA helicases, of which DDX41 is a member. There are 41 and 25 DEAD-box type RNA helicases in humans and budding yeast (*Saccharomyces cerevisiae*), respectively. They play multiple celluar roles including those involved with transcription, RNA splicing, ribosome biogenesis, and translation (32, 43, 44). RNA helicases are also regulators of genome stability (45). Single RNA helicases often play multiple roles, posing a challenge with regard to elucidation of the disease-relevant activities of the enzymes. DEAD-box type RNA helicases are named after a motif consisting of Asp-Glu-Ala-Asp (D-E-A-D) amino acids within RecA-like domain 1; they are often comparatively discussed with DEAH-box type RNA helicases (46). Although these RNA helicases both unwind RNA duplex or alter RNA-protein interactions *via* their ATPase activities, the molecular mechanisms employed to carry out this function are not shared by the two groups (47). Specifically, DEAD-box type RNA helicases recognize and unwind short RNA duplexes in an ATP-dependent manner, while DEAH-box type helicases form a tunnel through the RecA, Winged-helix (WH), Helix-bundle (HB) and Oligosaccharide-binding (OB) domains at their C-termini, where they translocate on the RNA by gripping the RNA in the tunnel. DEAH-box helicases may also alter the structure of RNA or the spliceosome by winding up RNA in a ‘winch-like’ manner (46). In the following sections, we discuss the roles of DDX41 that have been proposed in the literature to date.

## RNA splicing

**Figure 2:**
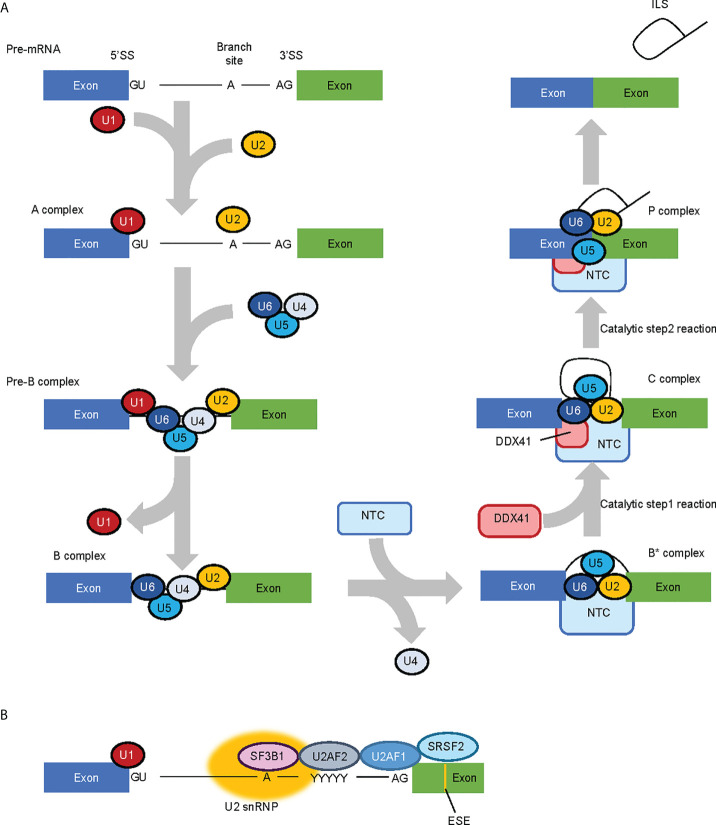
RNA splicing process and factors involved in the process. **(A)** Simplified RNA splicing process. The 5’ splice site (SS) and branch site of transcribed pre-mRNA are first recognized by U1 snRNP and U2 snRNP, respectively. U4/U6.U5 tri-snRNP is then recruited while U1 snRNP and U4 snRNP are released in a stepwise manner. The NineTeen Complex (NTC), consisting of 7 core NTC proteins and 14 NTC-associated proteins, is recruited to (and regulates the conformaiotn of) the spliceosome. In the catalytic Step 1 reaction, the 2’-OH of adenine in the branch site attacks the 5’SS and cleaves the RNA to form an intron lariat, and in the Step 2 reaction, the 3’-OH at the 3’ end of the free 5’ exon attacks the 3’SS and cleaves the RNA. DDX41 is likely recruited to the spliceosome at the C complex phase. **(B)** MDS-related RNA splicing factors. SF3B1 is involved in branch site recognition, U2AF1 in 3’ SS recognition and SRSF2 in exonic splicing enhancer (ESE) recognition, respectively. Therefore, most of the mutations in MDS-related RNA splicing factors are concentrated in factors involved in 3’SS recognition.

Comprehensive analysis for factors that constitute the spliceosome at each phase of RNA splicing have suggested that DDX41 is a component of splicing C complex (48, 49). RNA splicing occurs through two consecutive trans-esterification reactions (**Figure 2A**) (50); in the first process, the 2’-OH of adenine at the branch site attacks the 5’ splice site (SS) and cleaves the RNA, forming a 2’,5’-phosphodiester bond to create an intron lariat. This structure is called the C complex. In the second process, the 3’-OH at the 3’ end of the free 5’ exon attacks the 3’SS and cleaves the RNA, forming a phosphodiester bond between the 3’-OH terminus of the 5’-exon and the 5’-P terminus of the 3’-exon, leaving the intron lariat in the vicinity of the ligated exons. This structure is the P complex. RNA splicing involves five types of small nucleolar RNPs (snRNPs) (U1, U2, U4, U5 and U6 snRNP) and more than 100 proteins, which are required to carry out the structural transformation of pre-mRNA and spliceosome in a co-ordinated manner (50, 51).

Beyond DDX41, mutations in genes that encode RNA splicing-related factors implicated in myeloid malignancies are observed in about 40–60% of MDS patients (52, 53); of note, frequently mutated genes (namely, *SF3B1*, *SRSF2* and *U2AF1*) all encode factors involved in the recognition of the 3’SS (**Figure 2B**) (54). However, the nature of the RNA splicing aberrations are specific to each mutated splicing factor, rather than being shared between them all (55). DDX41 is incorporated into the spliceosome at the C complex when the SS has already been determined (49). Therefore, the role of DDX41 in RNA splicing will be largely different to that of typical MDS-related splicing factors. In fact, Li et al. showed that 21 of 176 cases with germline *DDX41* variants (with or without somatic *DDX41* mutations), and 2 of 19 cases with somatic *DDX41* mutations alone had mutations in at least one of the genes encoding typical MDS-related RNA splicing factors (SF3B1, SRSF2, U2AF1, U2AF2 and ZRSR2) with a variant allele frequency of 3% or more (9). Although clonal heterogeneity must be considered when discussing the co-existing mutations, these observations indicate that germline *DDX41* mutations are not necessarily mutually exclusive with mutations in these RNA splicing factors. Thus, the RNA splicing processes regulated by DDX41 may be distinct from those that are modulated by the other splicing factors.

Deletion of the *Caenorhabditis elegans* gene *sacy-1* (an ortholog of *DDX41*) has been reported to alter 3’SS selectivity (56). In contrast, little is known about the precise role of DDX41 in RNA splicing, although exon skipping was a major change feature of cells derived from AML patients with *DDX41* mutations (4), and splicing changes were also observed in hematopoietic progenitor cells from *Ddx41*-deficient mice, with exon skipping and retained introns being the major alterations (57).

## Recognition of nucleic acids from intracellular pathogens and induction of innate immune response by DDX41

In 2011, DDX41 was reported as a sensor that recognizes nucleic acids released from pathogens that invade the cytosol (58); the authors of this study found that knockdown of DDX41 diminished the induction of IFN-β following poly(dA:dT) stimulation. A subsequent study suggested that DDX41 recognizes cyclic di-guanosine monophosphate (c-di-GMP) (59), which is a cyclic di-nucleotide produced from two molecules of GTP by diguanylate cyclase that is widely used in bacteria as a second messenger for signal transduction (60). Upon recognition of these nucleic acids, DDX41 interacts with the adaptor molecule STING, which in turn triggers a STING-dependent innate immune response. DDX41 function is regulated by phosphorylation by Bruton’s tyrosine kinase (BTK), which mediates the interaction of DDX41 with STING by phosphorylating Y414 of DDX41 (61). The degradation of DDX41 is regulated by poly-ubiquitination (62); DDX41 interacts with the SPRY-PRY domain of TRIM21, an E3 ubiquitin ligase, *via* the DEADc domain, and TRIM21 appears to promote degradation of DDX41 *via* K48-linked polyubiquitination of K9 and K115.

**Figure 3:**
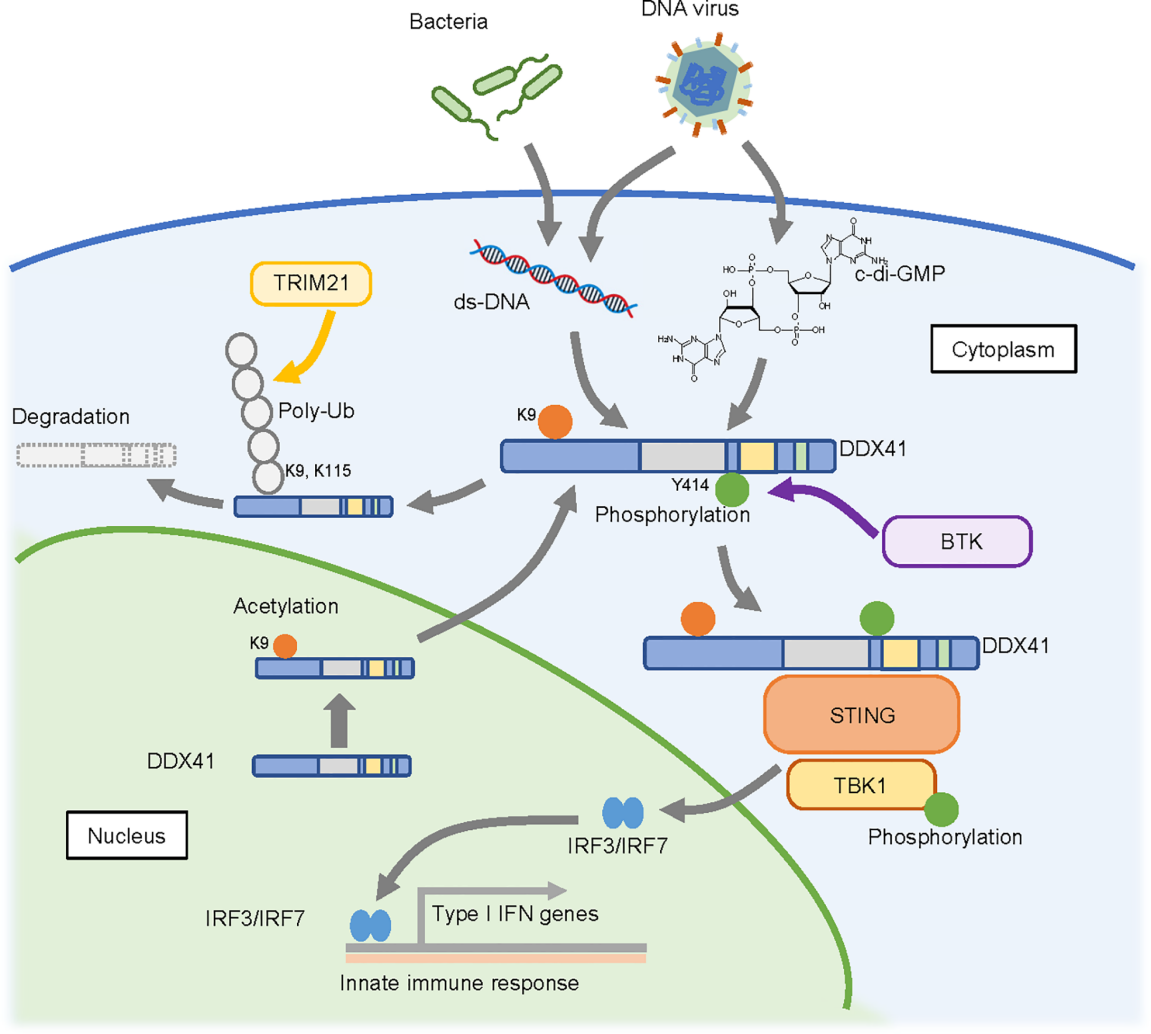
Possible cytosolic function of DDX41 as a nucleic acid sensor. DDX41 is exported to the cytoplasm in a nuclear localizing signal (NLS)-acetylation dependent manner and is activated by BTK-mediated phosphorylation. It senses double-strand DNA or c-di-GMP released from pathogens that invade the cytoplasm, and activates innate immune reactions through the STING-TBK1 axis. DDX41 is also reportedly degraded by TRIM21 mediated poly-ubiquitination (poly-Ub).

These reports suggest a role for DDX41 in promoting the innate immune response (**Figure 3**) (63). However, it remains unclear whether this function contributes to hematopoietic malignancies. Since germline variants of *DDX41* are likely loss-of-function, an assumption is that the immune response will be attenuated in cells expressing these variants. However, the opposite has also been reported, as the loss of DDX41 can induce R-loop formation (64, 65), which in turn leads to an inflammatory state in the cells. This is discussed in the next section.

DDX41 has a nuclear localization signal (NLS) at its N-terminus, and recent studies revealed that it is predominantly detected in the nucleus (66). However, there are at least two forms of DDX41 (33); one is a full length 70 kDa protein translated from the first methionine, and the other is a shorter 50 kDa form translated from the second methionine that lacks the NLS and is localized in the cytoplasm. It is possible that the shorter form is involved in nucleic acid sensing in the cytoplasm. On the other hand, there are two reports of DDX41 shuttling between the cytoplasm and nucleus (66, 67). In this context, our collaborators recently found that K9 acetylation of the NLS within DDX41 promotes its transport to the cytoplasm (68). They also suggested a possible mechanism by which the p.R525H mutant of DDX41 activates the innate immune response despite its attenuated helicase activity, as follows. RNA helicases generally have ATP-independent strand annealing activity, in addition to ATP-dependent strand unwinding activity. Since the p.R525H mutant exhibits less unwinding activity but retains annealing activity, it would effectively increase the amount of double-stranded cytosolic DNA available for activation of the STING-TBK1 pathway. The extent to which this influences hematopoietic malignancies remains unclear, as germline *DDX41* variants would not be expected to exhibit this selective retention of function.

## R-loop regulation by DDX41 limits DNA damage response signaling

**Figure 4:**
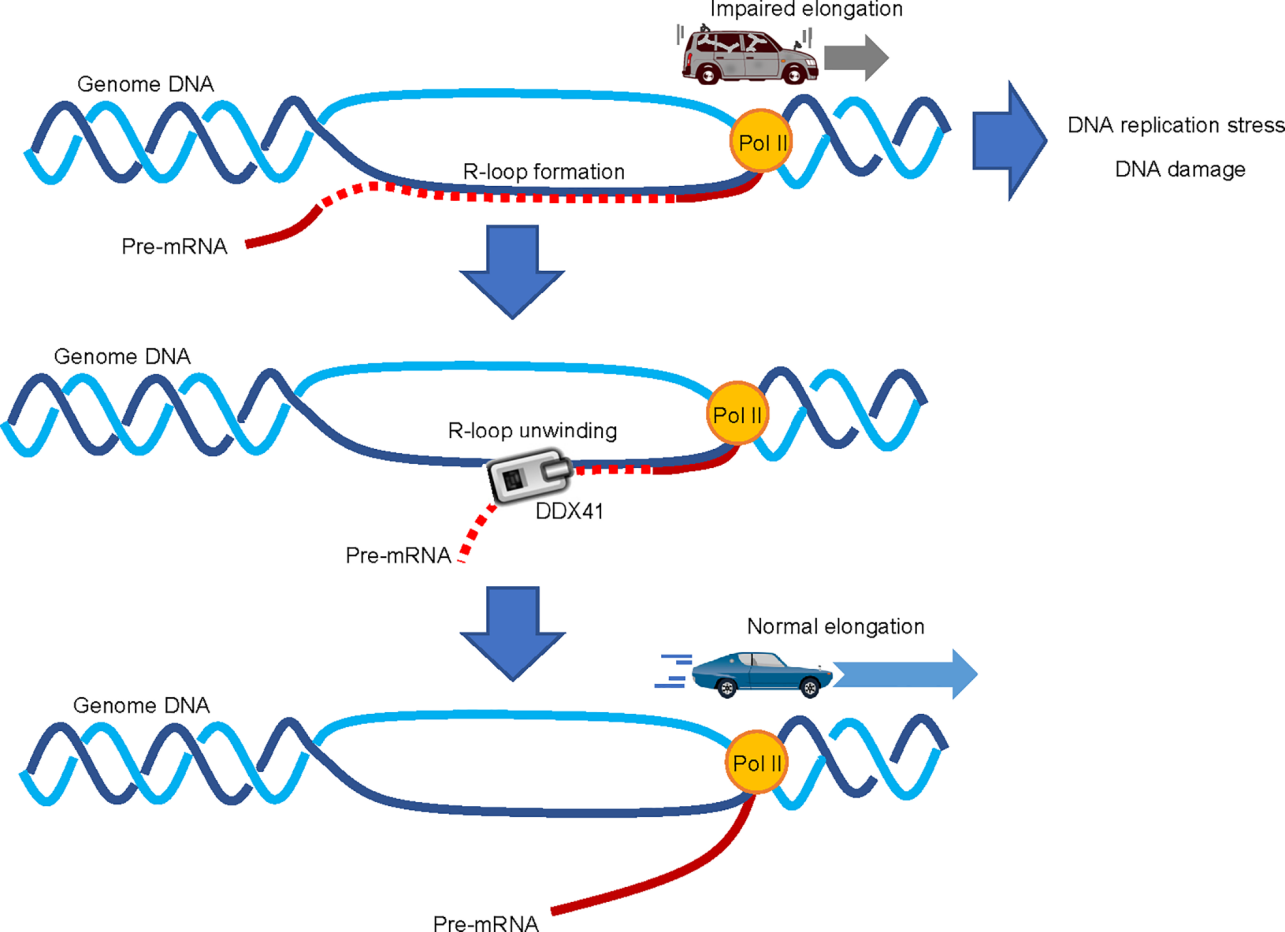
Involvement of DDX41 in R-loop resolution. Abnormal accumulation of R-loops impairs transcriptional elongation by RNA polymerase II (Pol II), which leads to increased replication stress and DNA damage; DDX41 has been proposed to directly unwind DNA : RNA hybrids to resolve R-loops.

An increase in R-loop formation occurs in MDS regardless of the mutation spectrum present in tumor cells (69, 70). R-loops are structures on genomic DNA consisting of DNA : RNA hybrids and single stranded DNA displaced from the paired strand (71) (**Figure 4**); they are involved in physiological processes such as transcription termination, immune globulin class-switching, mitochondrial DNA replication, and the DNA repair response (72). However, excessive accumulation of R-loops is associated with various pathological conditions, causing impaired transcriptional elongation and genomic instability. The first paper to report the involvement of R-loops in MDS showed that RNA splicing changes were exclusive to cells expressing different splicing factor mutants, while an increased DNA damage response and DNA replication stress were commonly observed (69). Although little is known about the process by which mutations in genes encoding RNA splicing factors lead to R-loop formation, SRSF2 can promote initiation of transcriptional elongation by releasing P-TEFb, a complex that activates RNA polymerase II (Pol II), by liberating it from an inhibitory factor (73). The presence of SRSF2 mutations in MDS cells may inhibit this effect and impair the pause-release of Pol II, which would render cells prone to R-loop formation (69). However, no mechanistic links between R-loop accumulation and mutations in genes encoding other MDS-related RNA splicing factors have been proposed. Further studies that will provide clearer insight are thus clearly warranted.

Two independent papers suggested that DDX41 regulates R-loop formation (39, 64). Expression of a Ddx41 loss-of-function mutant in zebrafish induces R-loop formation, along with the upregulation of inflammatory signals *via* the STING-TBK1 axis (65). The induction of inflammation upon DDX41 loss is somewhat contradictory to the aforementioned theory that DDX41 positively regulates inflammatory signals (63), but consistent with the fact that MDS and AML cells are often in an inflammatory state due to intrinsic production of inflammatory cytokines (74). As for the role of DDX41 in the regulation of R-loops, it is proposed that DDX41 directly resolves DNA : RNA hybrids (39). A comprehensive RNA-DNA proximity proteomics approach in the vicinity of R-loops identified several helicases, and showed that DDX41 knockdown induces DNA damage signaling. Since DDX41 can unwind DNA : RNA hybrids *in vitro* (68), it is plausible that DDX41 may indeed play a direct role in R-loop resolution. However, the mechanism by which DDX41 specifically resolves R-loops formed close to the transcription initiation site is largely unknown. While other molecules such as SENATAXIN, BLM, FANCM and WRN can unwind DNA : RNA hybrids (75), we await the outcome of future studies to delineate the specific role of DDX41 in the suppression of R-loop formation.

Increased R-loop formation is associated with activation of the DNA damage response (76). This includes activation of both Ataxia Telangiectasia Mutated (ATM) and Ataxia Telangiectasia and Rad3-related (ATR) pathways, which respond to double- and single-strand breaks, respectively (76). In particular, the ATR pathway has been implicated in the response to and resolution of replication stress caused by R-loop-induced collisions between transcription and replication. R-loops are also likely to induce double-strand breaks, which would be consistent with ATM activation. However, the mechanism by which this occurs is unclear, as the ATM pathway is activated by R-loops even in non-replicating cells (77). In any case, the ATM and ATR pathways could be potential therapeutic targets for tumor cells with accumulated R-loops. Indeed, the efficacy of Chk1 inhibitors for MDS with *SF3B1* mutations, as well as ATR inhibitors for those with *U2AF1* variants have been demonstrated (70, 78). Since hematopoietic malignancies with *DDX41* mutations also likely have increased levels of R-loops, inhibition of ATR/ATM may be a therapeutic option also for this disease.

## Involvement of DDX41 in ribosome biogenesis and translation

Our group has previously shown that DDX41 is involved in pre-ribosomal RNA (rRNA) processing (33). The study was inspired by findings from another group that (like many other nucleolar proteins) showed knockdown of DDX41 affected pre-rRNA processing (79). In ribosomal RNA synthesis in mammals, the 28S, 5.8S, and 18S rRNAs are transcribed together by RNA polymerase I to form 47S pre-rRNA, and 5S rRNA is transcribed by polymerase III; these pre-rRNAs undergo stepwise cleavage and trimming to produce mature rRNAs. In addition, small nucleolar ribonucleoproteins (snoRNPs) composed of small nucleolar RNAs (snoRNAs) and proteins undergo 2’-O-methylation and uridine isomerization (pseudouridylation) of rRNA (80). Finally, numerous ribosomal proteins that are bound to pre-rRNA promote the assembly of 60S and 40S ribosomal subunits. In our previous study, we revealed that the expression of p.R525H increased unprocessed 47S pre-rRNA and impaired ribosome biogenesis as a result. Although we have not yet identified the exact role that DDX41 plays in pre-rRNA processing process, we have found that the loss of DDX41 function causes a defect in pre-rRNA biogenesis and disrupts the balance of ribosome synthesis, thus leading to cell cycle arrest and apoptosis (33). Additionally, Chlon et al. reported that introduction of DDX41 mutation disrupted snoRNA processing, thereby leading to impaired ribosome function (81). snoRNAs are short RNAs of 60–300 nucleotides that localize in the nucleolus and are classified into two major groups (82). The first group contains Box C/D snoRNAs, which determine the position of 2’-O-methylation; the second is comprised of Box H/ACA snoRNAs, which are responsible for pseudouridylation. Each snoRNA interacts with proteins to form a small nucleolar ribonucleoprotein (snoRNP). For Box H/ACA snoRNA that are localized to the intronic regions of pre-mRNA, snoRNP-associated proteins first interact with the snoRNA region in the pre-mRNA, then intron lariats excised from the mRNA are processed into snoRNP (83). Cells deficient for DDX41 have impaired snoRNA processing (i.e., impaired isolation of snoRNA regions from introns), and this reduces ribosome biogenesis (81). Since RNA splicing is closely associated with snoRNA production, this study is noteworthy because it demonstrates a novel role for DDX41 in linking RNA splicing and ribosome biosynthesis. Abnormal expression of snoRNAs is implicated in disease phenotypes and drug resistance in hematopoietic malignancies and solid tumors (84–86), suggesting that snoRNAs play a significant role in the development of malignancies.

However, this discovery simultaneously raises another question regarding the role that DDX41 plays in snoRNA processing. Although reduced expression of DDX41 impairs intron removal in certain host genes, it is still unclear how it confers such splicing selectivity. Considering that RNA splicing factors play a role in snoRNA processing, as shown for IBP160 (87), it is possible that DDX41 in the splicing C complex is directly involved in snoRNA processing. Alternatively, snoRNPs and the spliceosome may interact *via* DDX41.

## Conclusion

Due to the limited variety and frequency of hematopoietic malignancies with gene mutations encoding RNA helicases, the link between these enzymes and oncogenesis has historically been unclear. However, this link has been partially elucidated by recent studies. Cells with *DDX41* mutations are more likely to exhibit a DNA damage phenotype that renders them more prone to apoptosis than to proliferation, at least prior to overt disease development. DDX41 may play a role in late process of RNA splicing, and mutant-related defects would affect the transcription and DNA replication that normally progress in concert with RNA splicing. This hypothesis may explain the unique phenotypes of this disease. Minor clones with biallelic *DDX41* mutations emerged from those with a germline variant and define the disease phenotype (81). However, no explanation has been provided for how such minor clones could contribute to the disease; it might be possible that cells with biallelic mutations create an inflammatory state through R-loop accumulation, or *via* other yet-unidentified mechanisms.

Myeloid malignancies with DDX41 variants respond relatively well to standard therapies using anthracycline and cytarabine with or without gemtuzumab ozogamicin for induction and consolidation treatments, or to hypomethylating agents for patients not eligible for intensive therapy. However, the disease is more likely to occur in the elderly and it is often difficult to administer cytotoxic chemotherapy to these patients. Therefore, treatments based on the concept of synthetic lethality with molecular targeted agents that inhibit DNA damage response pathways or RNA splicing processes could be a reasonable and promising approach.

## Author contributions

SS and HM wrote the manuscript. All authors contributed to the article and approved the submitted version.

## Funding

This study was supported by Japanese Society for the Promotion of Science Grants-in-Aid for Scientific Research No. 16H06279(PAGS), No. 21K08419, and No. 18K08334 (HM) and No. 22K10195 (SS). This work was also partly supported by the Foundation for Promotion of Cancer Research.

## Acknowledgments

The authors would like to express our sincere appreciation to all those who supported our work, especially Ms. Kazue Akita and Ms. Aya Higashi in our laboratory.

## Conflict of interest

The authors declare that the research was conducted in the absence of any commercial or financial relationships that could be construed as a potential conflict of interest.

## Publisher’s note

All claims expressed in this article are solely those of the authors and do not necessarily represent those of their affiliated organizations, or those of the publisher, the editors and the reviewers. Any product that may be evaluated in this article, or claim that may be made by its manufacturer, is not guaranteed or endorsed by the publisher.

## References

[1] Hou HA Tien HF. Genomic landscape in acute myeloid leukemia and its implications in risk classification and targeted therapies. J BioMed Sci (2020) 27:81. doi: 10.1186/s12929-020-00674-7 32690020 PMC7372828

[2] Palomo L Meggendorfer M Hutter S Twardziok S Ademà V Fuhrmann I. Molecular landscape and clonal architecture of adult myelodysplastic/myeloproliferative neoplasms. Blood (2020) 136:1851–62. doi: 10.1182/blood.2019004229 PMC7645608 32573691

[3] Rio-Machin A Vulliamy T Hug N Walne A Tawana K Cardoso S. The complex genetic landscape of familial MDS and AML reveals pathogenic germline variants. Nat Commun (2020) 11:1044. doi: 10.1038/s41467-020-14829-5 32098966 PMC7042299

[4] Polprasert C Schulze I Sekeres MA Makishima H Przychodzen B Hosono N. Inherited and somatic defects in DDX41 in myeloid neoplasms. Cancer Cell (2015) 27:658–70. doi: 10.1016/j.ccell.2015.03.017 PMC8713504 25920683

[5] Arber DA Orazi A Hasserjian R Thiele J Borowitz MJ Le Beau MM. The 2016 revision to the world health organization classification of myeloid neoplasms and acute leukemia. Blood (2016) 127:2391–405. doi: 10.1182/blood-2016-03-643544 27069254

[6] Andreou AZ. DDX41: a multifunctional DEAD-box protein involved in pre-mRNA splicing and innate immunity. Biol Chem (2021) 402:645–51. doi: 10.1515/hsz-2020-0367 33711218

[7] Jiang Y Zhu Y Qiu W Liu YJ Cheng G Liu ZJ. Structural and functional analyses of human DDX41 DEAD domain. Protein Cell (2017) 8:72–6. doi: 10.1007/s13238-016-0351-9 PMC5233616 27928732

[8] Cheah JJC Hahn CN Hiwase DK Scott HS Brown AL. Myeloid neoplasms with germline DDX41 mutation. Int J Hematol (2017) 106:163–74. doi: 10.1007/s12185-017-2260-y 28547672

[9] Li P Brown S Williams M White TA Xie W Cui W. The genetic landscape of germline DDX41 variants predisposing to myeloid neoplasms. Blood (2022)140:716–55. doi: 10.1182/blood.2021015135 PMC9389629 35671390

[10] Churpek JE Smith-Simmer K. DDX41-associated familial myelodysplastic syndrome and acute myeloid leukemia. In: GeneReviews. Seattle: University of Washington (2021). p. 1993–2022.

[11] Li P White T Xie W Cui W Peker D Zeng G. AML with germline DDX41 variants is a clinicopathologically distinct entity with an indolent clinical course and favorable outcome. Leukemia (2021) 36:664–74. doi: 10.1038/s41375-021-01404-0 34671111

[12] Alkhateeb HB Nanaa A Viswanatha D Foran JM Badar T Sproat L. Genetic features and clinical outcomes of patients with isolated and comutated DDX41-mutated myeloid neoplasms. Blood Adv (2022) 6:528–32. doi: 10.1182/bloodadvances.2021005738 PMC8791578 34644397

[13] Goyal T Tu ZJ Wang Z Cook JR. Clinical and pathologic spectrum of DDX41-mutated hematolymphoid neoplasms. Am J Clin Pathol (2021) 156:829–38. doi: 10.1093/ajcp/aqab027 33929502

[14] Qu S Li B Qin T Xu Z Pan L Hu N. Molecular and clinical features of myeloid neoplasms with somatic DDX41 mutations. Br J Haematol (2021) 192:1006–10. doi: 10.1111/bjh.16668 PMC9205684 32307695

[15] Sébert M Passet M Raimbault A Rahmé R Raffoux E Sicre de Fontbrune F. Germline DDX41 mutations define a significant entity within adult MDS/AML patients. Blood (2019) 134:1441–4. doi: 10.1182/blood.2019000909 31484648

[16] Maciejewski JP Padgett RA Brown AL Müller-Tidow C. DDX41-related myeloid neoplasia. Semin Hematol (2017) 54:94–7. doi: 10.1053/j.seminhematol.2017.04.007 PMC8190973 28637623

[17] Cardoso SR Ryan G Walne AJ Ellison A Lowe R Tummala H. Germline heterozygous DDX41 variants in a subset of familial myelodysplasia and acute myeloid leukemia. Leukemia (2016) 30:2083–6. doi: 10.1038/leu.2016.124 PMC5008455 27133828

[18] Duployez N Largeaud L Duchmann M Kim R Rieunier J Lambert J. Prognostic impact of DDX41 germline mutations in intensively treated acute myeloid leukemia patients: an ALFA-FILO study. Blood (2022) 40:756–68. doi: 10.1182/blood.2021015328 PMC9389637 35443031

[19] Opatz S Bamopoulos SA Metzeler KH Herold T Ksienzyk B Bräundl K. The clinical mutatome of core binding factor leukemia. Leukemia (2020) 34:1553–62. doi: 10.1038/s41375-019-0697-0 PMC7266744 31896782

[20] Faber ZJ Chen X Gedman AL Boggs K Cheng J Ma J. The genomic landscape of core-binding factor acute myeloid leukemias. Nat Genet (2016) 48:1551–6. doi: 10.1038/ng.3709 PMC5508996 27798625

[21] Rivera OD Mallory MJ Quesnel-Vallières M Chatrikhi R Schultz DC Carroll M. Alternative splicing redefines landscape of commonly mutated genes in acute myeloid leukemia. Proc Natl Acad Sci U.S.A. (2021) 118:e2014967118. doi: 10.1073/pnas.2014967118 33876749 PMC8054020

[22] Quesada AE Routbort MJ DiNardo CD Bueso-Ramos CE Kanagal-Shamanna R Khoury JD. DDX41 mutations in myeloid neoplasms are associated with male gender, TP53 mutations and high-risk disease. Am J Hematol (2019) 94:757–66. doi: 10.1002/ajh.25486 30963592

[23] Iacobucci I Wen J Meggendorfer M Choi JK Shi L Pounds SB. Genomic subtyping and therapeutic targeting of acute erythroleukemia. Nat Genet (2019) 51:694–704. doi: 10.1038/s41588-019-0375-1 30926971 PMC6828160

[24] Lewinsohn M Brown AL Weinel LM Phung C Rafidi G Lee MK. Novel germ line DDX41 mutations define families with a lower age of MDS/AML onset and lymphoid malignancies. Blood (2016) 127:1017–23. doi: 10.1182/blood-2015-10-676098 PMC4968341 26712909

[25] Shin WY Yoon SY Park R Kim JA Song HH Bang HI. A novel bi-alleleic DDX41 mutations in b-cell lymphoblastic leukemia: Case report. BMC Med Genomics (2022) 15:46. doi: 10.1186/s12920-022-01191-2 35246110 PMC8897883

[26] Singhal D Hahn CN Feurstein S Wee LYA Moma L Kutyna MM. Targeted gene panels identify a high frequency of pathogenic germline variants in patients diagnosed with a hematological malignancy and at least one other independent cancer. Leukemia (2021) 35:3245–56. doi: 10.1038/s41375-021-01246-w 33850299

[27] Bannon SA Routbort MJ Montalban-Bravo G Mehta RS Jelloul FZ Takahashi K. Next-generation sequencing of DDX41 in myeloid neoplasms leads to increased detection of germline alterations. Front Oncol (2020) 10:582213. doi: 10.3389/fonc.2020.582213 33585199 PMC7878971

[28] Fazio F Quintini M Carmosino I Matteucci C Miulli E Pellanera F. New dead/H-box helicase gene (ddx41) mutation in an Italian family with recurrent leukemia. Leuk Lymph (2021) 62:2280–3. doi: 10.1080/10428194.2021.1910689 33836623

[29] Polprasert C Takeda J Niparuck P Rattanathammethee T Pirunsarn A Suksusut A. Novel DDX41 variants in Thai patients with myeloid neoplasms. Int J Hematol (2020) 111:241–6. doi: 10.1007/s12185-019-02770-3 31713024

[30] Király AP Kállay K Gángó A Kellner Á Egyed M Szőke A. Familial acute myeloid leukemia and myelodysplasia in Hungary. Pathol Oncol Res (2018) 24:83–8. doi: 10.1007/s12253-017-0216-4 28357685

[31] Choi EJ Cho YU Hur EH Jang S Kim N Park HS. Unique ethnic features of DDX41 mutations in patients with idiopathic cytopenia of undetermined significance, myelodysplastic syndrome, or acute myeloid leukemia. Haematologica (2022) 107:510–8. doi: 10.3324/haematol.2020.270553 PMC8804579 33626862

[32] Linder P Jankowsky E. From unwinding to clamping - the DEAD box RNA helicase family. Nat Rev Mol Cell Biol (2011) 12:505–16. doi: 10.1038/nrm3154 21779027

[33] Kadono M Kanai A Nagamachi A Shinriki S Kawata J Iwato K. Biological implications of somatic DDX41 p.R525H mutation in acute myeloid leukemia. Exp Hematol (2016) 44:745–54.e4. doi: 10.1016/j.exphem.2016.04.017 27174803

[34] Choi EJ Cho YU Hur EH Park HS Choi Y Lee JH. Clinical implications and genetic features of clonal cytopenia of undetermined significance compared to lower-risk myelodysplastic syndrome. Br J Haematol (2022) 198:703–12. doi: 10.1111/bjh.18273 35612271

[35] Gibson CJ Kim HT Zhao L Murdock HM Hambley B Ogata A. Donor clonal hematopoiesis and recipient outcomes after transplantation. J Clin Oncol (2022) 40:189–201. doi: 10.1200/jco.21.02286 34793200 PMC8718176

[36] Kobayashi S Kobayashi A Osawa Y Nagao S Takano K Okada Y. Donor cell leukemia arising from preleukemic clones with a novel germline DDX41 mutation after allogenic hematopoietic stem cell transplantation. Leukemia (2017) 31:1020–2. doi: 10.1038/leu.2017.44 28194039

[37] Williams L Doucette K Karp JE Lai C. Genetics of donor cell leukemia in acute myelogenous leukemia and myelodysplastic syndrome. Bone Marrow Transplant (2021) 56:1535–49. doi: 10.1038/s41409-021-01214-z 33686252

[38] Berger G van den Berg E Sikkema-Raddatz B Abbott KM Sinke RJ Bungener LB. Re-emergence of acute myeloid leukemia in donor cells following allogeneic transplantation in a family with a germline DDX41 mutation. Leukemia (2017) 31:520–2. doi: 10.1038/leu.2016.310 27795557

[39] Mosler T Conte F Longo GMC Mikicic I Kreim N Möckel MM. R-loop proximity proteomics identifies a role of DDX41 in transcription-associated genomic instability. Nat Commun (2021) 12:7314. doi: 10.1038/s41467-021-27530-y 34916496 PMC8677849

[40] Abou Dalle I Kantarjian H Bannon SA Kanagal-Shamanna R Routbort M Patel KP. Successful lenalidomide treatment in high risk myelodysplastic syndrome with germline DDX41 mutation. Am J Hematol (2020) 95:227–9. doi: 10.1002/ajh.25610 31400013

[41] Negoro E Radivoyevitch T Polprasert C Adema V Hosono N Makishima H. Molecular predictors of response in patients with myeloid neoplasms treated with lenalidomide. Leukemia (2016) 30:2405–9. doi: 10.1038/leu.2016.228 PMC5143200 27560106

[42] Fairman-Williams ME Guenther UP Jankowsky E. SF1 and SF2 helicases: family matters. Curr Opin Struct Biol (2010) 20:313–24. doi: 10.1016/j.sbi.2010.03.011 PMC2916977 20456941

[43] Pan C Russell R. Roles of DEAD-box proteins in RNA and RNP folding. RNA Biol (2010) 7:667–76. doi: 10.4161/rna.7.6.13571 PMC3073326 21045543

[44] Ali MAM. The DEAD-box protein family of RNA helicases: sentinels for a myriad of cellular functions with emerging roles in tumorigenesis. Int J Clin Oncol (2021) 26:795–825. doi: 10.1007/s10147-021-01892-1 33656655

[45] Cargill M Venkataraman R Lee S. DEAD-box RNA helicases and genome stability. Genes (Basel) (2021) 12:1471. doi: 10.3390/genes12101471 34680866 PMC8535883

[46] De Bortoli F Espinosa S Zhao R. DEAH-box RNA helicases in pre-mRNA splicing. Trends Biochem Sci (2021) 46:225–38. doi: 10.1016/j.tibs.2020.10.006 PMC8112905 33272784

[47] Gilman B Tijerina P Russell R. Distinct RNA-unwinding mechanisms of DEAD-box and DEAH-box RNA helicase proteins in remodeling structured RNAs and RNPs. Biochem Soc Trans (2017) 45:1313–21. doi: 10.1042/bst20170095 PMC5960804 29150525

[48] Wahl MC Will CL Lührmann R. The spliceosome: Design principles of a dynamic RNP machine. Cell (2009) 136:701–18. doi: 10.1016/j.cell.2009.02.009 19239890

[49] Cvitkovic I Jurica MS. Spliceosome database: a tool for tracking components of the spliceosome. Nucleic Acids Res (2013) 41:D132–41. doi: 10.1093/nar/gks999 PMC3531166 23118483

[50] Wilkinson ME Charenton C Nagai K. RNA Splicing by the spliceosome. Annu Rev Biochem (2020) 89:359–88. doi: 10.1146/annurev-biochem-091719-064225 31794245

[51] Will CL Lührmann R. Spliceosome structure and function. Cold Spring Harb Perspect Biol (2011) 3:a003707. doi: 10.1101/cshperspect.a003707 21441581 PMC3119917

[52] Haferlach T Nagata Y Grossmann V Okuno Y Bacher U Nagae G. Landscape of genetic lesions in 944 patients with myelodysplastic syndromes. Leukemia (2014) 28:241–7. doi: 10.1038/leu.2013.336 PMC3918868 24220272

[53] Yoshida K Sanada M Shiraishi Y Nowak D Nagata Y Yamamoto R. Frequent pathway mutations of splicing machinery in myelodysplasia. Nature (2011) 478:64–9. doi: 10.1038/nature10496 21909114

[54] Anczuków O Krainer AR. Splicing-factor alterations in cancers. RNA (2016) 22:1285–301. doi: 10.1261/rna.057919.116 PMC4986885 27530828

[55] Hershberger CE Moyer DC Adema V Kerr CM Walter W Hutter S. Complex landscape of alternative splicing in myeloid neoplasms. Leukemia (2021) 35:1108–20. doi: 10.1038/s41375-020-1002-y PMC8101081 32753690

[56] Tsukamoto T Gearhart MD Kim S Mekonnen G Spike CA Greenstein D. Insights into the involvement of spliceosomal mutations in myelodysplastic disorders from analysis of SACY-1/DDX41 in caenorhabditis elegans. Genetics (2020) 214:869–93. doi: 10.1534/genetics.119.302973 PMC7153925 32060018

[57] Ma J Mahmud N Bosland MC Ross SR. DDX41 is needed for pre- and postnatal hematopoietic stem cell differentiation in mice. Stem Cell Rep (2022) 17:879–93. doi: 10.1016/j.stemcr.2022.02.010 PMC9023775 35303436

[58] Zhang Z Yuan B Bao M Lu N Kim T Liu YJ. The helicase DDX41 senses intracellular DNA mediated by the adaptor STING in dendritic cells. Nat Immunol (2011) 12:959–65. doi: 10.1038/ni.2091 PMC3671854 21892174

[59] Parvatiyar K Zhang Z Teles RM Ouyang S Jiang Y Iyer SS. The helicase DDX41 recognizes the bacterial secondary messengers cyclic di-GMP and cyclic di-AMP to activate a type I interferon immune response. Nat Immunol (2012) 13:1155–61. doi: 10.1038/ni.2460 PMC3501571 23142775

[60] Krasteva PV Giglio KM Sondermann H. Sensing the messenger: the diverse ways that bacteria signal through c-di-GMP. Protein Sci (2012) 21:929–48. doi: 10.1002/pro.2093 PMC3403432 22593024

[61] Lee KG Kim SS Kui L Voon DC Mauduit M Bist P. Bruton's tyrosine kinase phosphorylates DDX41 and activates its binding of dsDNA and STING to initiate type 1 interferon response. Cell Rep (2015) 10:1055–65. doi: 10.1016/j.celrep.2015.01.039 25704810

[62] Zhang Z Bao M Lu N Weng L Yuan B Liu YJ. The E3 ubiquitin ligase TRIM21 negatively regulates the innate immune response to intracellular double-stranded DNA. Nat Immunol (2013) 14:172–8. doi: 10.1038/ni.2492 PMC3645272 23222971

[63] Jiang Y Zhu Y Liu ZJ Ouyang S. The emerging roles of the DDX41 protein in immunity and diseases. Protein Cell (2017) 8:83–9. doi: 10.1007/s13238-016-0303-4 PMC5291771 27502187

[64] Weinreb JT Ghazale N Pradhan K Gupta V Potts KS Tricomi B. Excessive r-loops trigger an inflammatory cascade leading to increased HSPC production. Dev Cell (2021) 56:627–40. doi: 10.1016/j.devcel.2021.02.006 PMC8258699 33651979

[65] Weinreb JT Gupta V Sharvit E Weil R Bowman TV. Ddx41 inhibition of DNA damage signaling permits erythroid progenitor expansion in zebrafish. Haematologica (2021) 107:644–54. doi: 10.3324/haematol.2020.257246 PMC8883538 33763998

[66] Ma JX Li JY Fan DD Feng W Lin AF Xiang LX. Identification of DEAD-box RNA helicase DDX41 as a trafficking protein that involves in multiple innate immune signaling pathways in a zebrafish model. Front Immunol (2018) 9:1327. doi: 10.3389/fimmu.2018.01327 29942316 PMC6005158

[67] Abdul-Ghani M Hartman KL Ngsee JK. Abstrakt interacts with and regulates the expression of sorting nexin-2. J Cell Physiol (2005) 204:210–8. doi: 10.1002/jcp.20285 PMC2963638 15690390

[68] Singh RS Vidhyasagar V Yang S Arna AB Yadav M Aggarwal A. DDX41 is required for cGAS-STING activation against DNA virus infection. Cell Rep (2022) 39:110856. doi: 10.1016/j.celrep.2022.110856 35613581 PMC9205463

[69] Chen L Chen JY Huang YJ Gu Y Qiu J Qian H. The augmented r-loop is a unifying mechanism for myelodysplastic syndromes induced by high-risk splicing factor mutations. Mol Cell (2018) 69:412–25.e6. doi: 10.1016/j.molcel.2017.12.029 29395063 PMC5957072

[70] Singh S Ahmed D Dolatshad H Tatwavedi D Schulze U Sanchi A. SF3B1 mutations induce r-loop accumulation and DNA damage in MDS and leukemia cells with therapeutic implications. Leukemia (2020) 34:2525–30. doi: 10.1038/s41375-020-0753-9 PMC7449882 32076118

[71] Allison DF Wang GG. R-loops: formation, function, and relevance to cell stress. Cell Stress (2019) 3:38–46. doi: 10.15698/cst2019.02.175 31225499 PMC6551709

[72] Niehrs C Luke B. Regulatory r-loops as facilitators of gene expression and genome stability. Nat Rev Mol Cell Biol (2020) 21:167–78. doi: 10.1038/s41580-019-0206-3 PMC7116639 32005969

[73] Ji X Zhou Y Pandit S Huang J Li H Lin CY. SR proteins collaborate with 7SK and promoter-associated nascent RNA to release paused polymerase. Cell (2013) 153:855–68. doi: 10.1016/j.cell.2013.04.028 PMC4103662 23663783

[74] Soyfer EM Fleischman AG. Inflammation in myeloid malignancies: From bench to bedside. J Immunother Precis Oncol (2021) 4:160–7. doi: 10.36401/jipo-21-3 PMC9138438 35663100

[75] Rinaldi C Pizzul P Longhese MP Bonetti D. Sensing r-Loop-Associated DNA damage to safeguard genome stability. Front Cell Dev Biol (2020) 8:618157. doi: 10.3389/fcell.2020.618157 33505970 PMC7829580

[76] Barroso S Herrera-Moyano E Muñoz S García-Rubio M Gómez-González B Aguilera A. The DNA damage response acts as a safeguard against harmful DNA-RNA hybrids of different origins. EMBO Rep (2019) 20:e47250. doi: 10.15252/embr.201847250 31338941 PMC6726908

[77] Tresini M Warmerdam DO Kolovos P Snijder L Vrouwe MG Demmers JA. The core spliceosome as target and effector of non-canonical ATM signalling. Nature (2015) 523:53–8. doi: 10.1038/nature14512 PMC4501432 26106861

[78] Nguyen HD Leong WY Li W Reddy PNG Sullivan JD Walter MJ. Spliceosome mutations induce r loop-associated sensitivity to ATR inhibition in myelodysplastic syndromes. Cancer Res (2018) 78:5363–74. doi: 10.1158/0008-5472.can-17-3970 PMC6139047 30054334

[79] Tafforeau L Zorbas C Langhendries JL Mullineux ST Stamatopoulou V Mullier R. The complexity of human ribosome biogenesis revealed by systematic nucleolar screening of pre-rRNA processing factors. Mol Cell (2013) 51:539–51. doi: 10.1016/j.molcel.2013.08.011 23973377

[80] Weinstein LB Steitz JA. Guided tours: from precursor snoRNA to functional snoRNP. Curr Opin Cell Biol (1999) 11:378–84. doi: 10.1016/s0955-0674(99)80053-2 10395551

[81] Chlon TM Stepanchick E Hershberger CE Daniels NJ Hueneman KM Kuenzi Davis A. Germline DDX41 mutations cause ineffective hematopoiesis and myelodysplasia. Cell Stem Cell (2021) 28:1966–81. doi: 10.1016/j.stem.2021.08.004 PMC8571055 34473945

[82] Wajahat M Bracken CP Orang A. Emerging functions for snoRNAs and snoRNA-derived fragments. Int J Mol Sci (2021) 22:10193. doi: 10.3390/ijms221910193 34638533 PMC8508363

[83] Kufel J Grzechnik P. Small nucleolar RNAs tell a different tale. Trends Genet (2019) 35:104–17. doi: 10.1016/j.tig.2018.11.005 30563726

[84] Lin LM Pan Q Sun YM Wang WT. Small nucleolar RNA is potential as a novel player in leukemogenesis and clinical application. Blood Sci (2021) 3:122–31. doi: 10.1097/bs9.0000000000000091 PMC8975097 35402848

[85] Liang J Wen J Huang Z Chen XP Zhang BX Chu L. Small nucleolar RNAs: Insight into their function in cancer. Front Oncol (2019) 9:587. doi: 10.3389/fonc.2019.00587 31338327 PMC6629867

[86] Fazi F Fatica A. Regulation of ribosome function by RNA modifications in hematopoietic development and leukemia: It is not only a matter of m(6)A. Int J Mol Sci (2021) 22:4755. doi: 10.3390/ijms22094755 33946178 PMC8125340

[87] Hirose T Ideue T Nagai M Hagiwara M Shu MD Steitz JA. A spliceosomal intron binding protein, IBP160, links position-dependent assembly of intron-encoded box C/D snoRNP to pre-mRNA splicing. Mol Cell (2006) 23:673–84. doi: 10.1016/j.molcel.2006.07.011 16949364

